# Balance in Blind Subjects: Cane and Fingertip Touch Induce Similar Extent and Promptness of Stance Stabilization

**DOI:** 10.3389/fnins.2018.00639

**Published:** 2018-09-11

**Authors:** Stefania Sozzi, Francesco Decortes, Monica Schmid, Oscar Crisafulli, Marco Schieppati

**Affiliations:** ^1^Centro Studi Attività Motorie, Istituti Clinici Scientifici Maugeri, Pavia, Italy; ^2^Centro di Riabilitazione Visiva, Istituti Clinici Scientifici Maugeri, Pavia, Italy; ^3^Department of Neurosciences, Rehabilitation, Ophthalmology, Genetics and Maternal Child Health, University of Genoa, Genoa, Italy; ^4^Department of Exercise and Sport Science, LUNEX International University of Health, Exercise and Sports, Differdange, Luxembourg

**Keywords:** haptics, cane, finger touch, blindness, stance control

## Abstract

Subjects with low vision often use a cane when standing and walking autonomously in everyday life. One aim of this study was to assess differences in the body stabilizing effect produced by the contact of the cane with the ground or by the fingertip touch of a firm surface. Another aim was to estimate the promptness of balance stabilization (or destabilization) on adding (or withdrawing) the haptic input from cane or fingertip. Twelve blind subjects and two subjects with severe visual impairment participated in two experimental protocols while maintaining the tandem Romberg posture on a force platform. In one protocol, subjects lowered the cane to a second platform on the ground and lifted it in sequence at their own pace. In the other protocol, they touched an instrumented pad with the index finger and withdrew the finger from the pad in sequence. In both protocols, subjects were asked to exert a force not granting mechanical stabilization. Under steady-state condition, the finger touch or the contact of the cane with the ground significantly reduced (to ∼78% and ∼86%, respectively) the amplitude of medio-lateral oscillation of the centre of foot pressure (CoP). Oscillation then increased when haptic information was removed. The delay to the change in body oscillation after the haptic shift was longer for addition than withdrawal of the haptic information (∼1.4 s and ∼0.7 s, respectively; *p* < 0.001), but was not different between the two haptic conditions (finger and cane). Similar stabilizing effects of input from cane on the ground and from fingertip touch, and similar latencies to integrate haptic cue from both sources, suggest that the process of integration of the input for balance control is initiated by the haptic stimulus at the interface cane-hand. Use of a tool is as helpful as the fingertip input, and does not produce different stabilization. Further, the latencies to haptic cue integration (from fingertip or cane) are similar to those previously found in a group of sighted subjects, suggesting that integration delays for automatic balance stabilization are not modified by visual impairment. Haptic input from a tool is easily exploited by the neural circuits subserving automatic balance stabilization in blind people, and its use should be enforced by sensory-enhancing devices and appropriate training.

## Introduction

Subjects with visual impairment often use a cane to move autonomously in everyday life (Jeka, 1997). The cane helps to partially overcome the mobility restriction associated with low vision, by assisting subjects in detecting obstacles along their path thereby decreasing the risk of falling (White et al., 2015), by conferring them a sense of stability (Bateni and Maki, 2005; Virgili and Rubin, 2010) and by providing a haptic cue to help estimate heading direction and orientation of the body in the gravitational field (Lederman and Klatzky, 2009; Lacquaniti et al., 2015).

Pioneering studies by Holden et al. (1994), Jeka and Lackner (1994), and Jeka et al. (1996) in sighted subjects showed that the simple contact by a finger with a solid frame is as effective as the sight of the surrounding environment in reducing postural sway when compared to no contact, eyes closed condition. Remarkably, the force levels exerted by the finger or by the cane were below those necessary to provide significant mechanical stabilization. Several studies followed, and all arrived at the conclusion that a light fingertip touch improves the control of standing by providing additional somatosensory information (Clapp and Wing, 1999; Baldan et al., 2014; Honeine and Schieppati, 2014; Schieppati et al., 2014; Bernard-Demanze et al., 2015; Honeine et al., 2015). Contact of a cane with the ground has been shown to be similarly effective in reducing postural sway in both sighted subjects and in small cohorts of non-sighted subjects as well (Jeka et al., 1996; Albertsen et al., 2010).

Sway stabilization must be rapid, in order to promptly adapt balance control to the haptic supplementation. The time that elapses from the instant of the haptic cue to postural stabilization is a critical period, during which the nervous system integrates the additional sensory input and prepares to shift to the new ‘postural set.’ This time period would be especially important in visually impaired subjects during their daily life activities. The latency of the postural resetting in response to the new haptic input from fingertip touch was estimated in sighted subjects standing with eyes closed (Rabin et al., 2006; Sozzi et al., 2011, 2012) and found to be in the order of 1 s. This was considered the time necessary for the integration and reweighting of the new information by the circuits responsible for balance control (Honeine et al., 2015). Conversely, the time period elapsing from the withdrawal of the finger touch to the increase in body sway was significantly shorter than that observed on addition of the haptic input. The latencies to the changes in CoP oscillation after the contact of a cane to the ground (or its removal) were estimated in a group of young sighted subjects (Sozzi et al., 2017). These latencies were similar to those occurring on fingertip touch, suggesting a substantial equivalence of the haptic information derived from “direct” fingertip contact and “indirect” cane contact with the floor. The latencies to fingertip light touch had been also estimated in a small population of blind subjects (Schieppati et al., 2014). They proved to be broadly similar to those of sighted people, except in a few congenital blind subjects that appeared to react early to the haptic finger cue.

Aim of the present investigation was: (a) to assess in a new population of blind subjects the effect on body sway of the haptic sensory input produced by the contact of a cane with the ground in stabilizing the standing body, and (b) to compare these effects to those produced by the light finger touch in the same cohort. Moreover, in the assumption that a prompt reaction to the haptic input would be a priority, we wanted (c) to estimate and compare in this same cohort the time necessary to achieve stabilization or destabilization, on adding or withdrawing the haptic input from either cane or finger touch.

In the background of this study stays the issue of the capacity of the blind subjects to exploit haptic inputs from a cane as an effective aid to reach balance stabilization and maintain equilibrium. Do subjects with severe visual impairment have an edge by using the cane compared to using a finger? Or does appropriate cane use require a higher level of conscious control than the finger touching task (Honeine et al., 2017) or larger anticipatory postural adjustments (Chabran et al., 2001) that might attenuate the incoming input (Starr and Cohen, 1985) and interfere with the promptness of stabilization?

Further, the two haptic inputs would depend on the activation of separate receptors and travel through separate central pathways; the distance of the supports from the body is different, and cane input from a distant point may have different effects on postural steadiness than the finger has, since light touch may be integrated as the horizontal distance between body center of mass and the haptic surface (Assländer et al., 2018); the preparation of the reaching movement to catch either of the two stable structures, and its control, would require a distinct set of postural adjustments as well (Aruin and Latash, 1996; Castellote et al., 2004; Krishnan et al., 2012) potentially leading to context-dependent modulation of tactile input (Juravle et al., 2013); different arm orientations and task features might produce changes in arm proprioception and cutaneous feedback (Rabin et al., 1999; Chapman and Beauchamp, 2006); and the difference in the features of the cutaneous stimuli may produce different cortical activation (Kojima et al., 2018). As a consequence, the integration and weighting of the two haptic inputs (direct with the finger, indirect with the cane) might produce a different degree of stabilization or need a different time interval before the stabilizing effects on balance are fully expressed. Further, the information from the cane contact with the ground would be processed by the blind subjects in a different way than that from the finger touch, because the cane also conveys some information about the free open space around them and about their orientation in that space, or because cross-modal brain plasticity might have differently affected the tactile and haptic sensory channel (Kupers et al., 2006; Fiehler and Rösler, 2010; see for a review, Parreira et al., 2017).

## Materials and Methods

### Subjects

Twelve blind subjects (according to the ICD-10 classification, with visual acuity < 1/20) and two subjects with severe visual impairment (visual acuity < 2/10) participated in this study (**Table 1**). Their mean (±SD) age, weight and height were 47.1 years ± 14.8, 81.4 kg ± 21.9 and 171.1 cm ± 9.2. The blindness was of varied etiology. Three of the subjects had severe visual impairment from infancy (early, age <5 years), eleven had vision problems starting later in life (late, age >5 years) and became gradually blind still later. All of them had received an orienteering and training course except three, which were receiving the course at the time of their recruitment in the study.

**Table 1:** Characteristics of the participants.

Subject/sex	Diagnosis	Visual acuity	Age at diagnosis/cane user for # years
1/F	Age-related macular degeneration	0, ability to tell light from dark	Late/7
2/M	Aniridia and nystagmus	1/20	Early/1
3/M	Retinopathy of prematurity	0, ability to tell light from dark	Early/8
4/M	Cortical blindness (multifocal leukoencephalopathy in AIDS)	0	Late/2
5/F	Stargardt macular degeneration	1/50	Late/4
6/M	Retinitis pigmentosa	2/10	Late/2
7/F	Leber congenital amaurosis	0	Early/11
8/M	Congenital glaucoma	0	Late/30
9/M	Angioblastoma with ocular nerves damage	0, ability to tell light from dark	Late/30
10/F	Meningioma of olfactory groove	0, ability to tell light from dark	Late/12
11/F	Tapetoretinal degeneration	RE: 1/50, LE: 1/20	Early/0
12/M	Age-related macular degeneration and glaucoma	1/20	Late/0
13/M	Bilateral retinal detachment and glaucoma	0, ability to tell light from dark	Late/10
14/M	Stargardt macular degeneration	RE 1/10, LE: counting finger at 30 cm distance	Late/0


*Early, <5 years; late, >5 years; RE, right eye; LE, left eye.*

All subjects were naïve to the experimental task and had not participated previously in balance control investigations. All of them, before participating in the experiments, signed the informed consent form approved by the ethics committee of the Istituti Clinici Scientifici Maugeri (# 757 CEC) in accordance with the Declaration of Helsinki.

**FIGURE 1:**
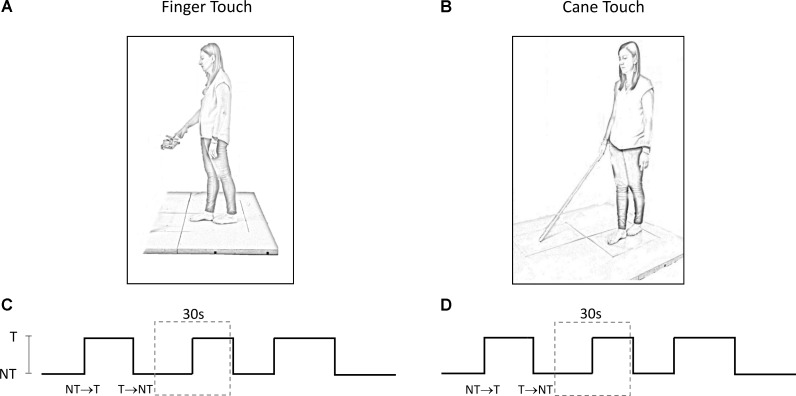
**(A,B)** Subject stood with feet in tandem position on a force platform and performed a series of trials under ‘Finger touch’ **(A)** and ‘Cane touch’ condition **(B)**. **(C,D)** The duration of the time intervals that contained the haptic changes varied randomly from 15 s to 25 s (three such periods are shown). For the analysis, each acquisition epoch was then divided in periods of 30 s duration (dotted rectangles) centered on the change in haptic condition.

The experiments took place in a normally lit room. Subjects participated in two different experimental protocols, performed in two sessions taking place in different days, maintaining the tandem Romberg posture with eyes closed (EC) on a force platform, with the great toe of the rear foot just behind the heel of the front foot (**Figure 1**). Seven subjects chose the right foot as the rear foot. This posture was utilized in order to magnify the medio-lateral (M-L) sway variations connected to the changes in haptic information (Sozzi et al., 2011, 2012, 2013; Honeine et al., 2015). For the entire duration of the experiments, an operator stood near the subjects in order to help them if they lost balance. This never happened and no subject even lifted a foot during the trial. The overall duration of each session (periods of rest included) lasted about 2 h.

### Centre of Foot Pressure (CoP) Oscillation

Platforms force (Kistler 9286BA) signals were acquired at 140 Hz (SMART-D system, BTS, Italy). The output of the platform was the instantaneous position of the centre of foot pressure (CoP) along the sagittal (antero-posterior, A-P) and the frontal plane (M-L). To quantify the amplitude of the CoP oscillations, the CoP M-L and A-P position traces were high-pass filtered with a second order Butterworth filter (cut-off frequency 0.1 Hz) and rectified with a software developed in Labview (National Instruments, United States) before averaging. No low pass filtering was applied.

### Finger Touch

Subjects slowly flexed the right hand to lightly touch a pad with the tip of the index finger, or to withdraw the finger from the pad, after a verbal go-signal given by the operator. Subjects were asked not to move in a reaction-time mode on hearing the verbal signal, but to self-pace the finger movement when ready. The touch-pad was horizontally oriented and positioned in front of the subject’s right hemi-body at about the height of the flexed forearm (**Figure 1A**). In order to facilitate the light touch of the pad, the height of the touch pad was adjusted for each subject. The touch-pad was instrumented with a strain gauge. The signal recorded from the strain gauge was stored in a PC and then utilized to detect the time at which the touch occurred or the finger was removed from the pad, and to calculate the force applied on the pad. A few practice trials were run to obtain touch forces on the pad smaller than 1 N (Jeka and Lackner, 1994). These touching and withdrawing tasks were repeated in sequence. The time intervals between each movement varied pseudo-randomly between 15 s and 25 s.

### Cane Touch

Subjects held with their dominant hand (right hand for 13 subjects) a straight plastic cane of 1 m length and 100 g weight (**Figure 1B**). Except for this, the protocol mirrored the ‘finger-touch’ protocol described above. The force applied by the cane on the ground and the time instant of the contact of the cane with the ground (or of cane lifting) were identified from the force signal recorded by a second force platform. This platform was placed in front of the subject and laterally spaced from the platform on which the subject stood (there were 55 cm from the center of the first platform to the center of the second platform). Again, subjects were asked not to move the cane in a reaction-time mode on hearing the verbal signal, but to self-pace the movement necessary for lowering or lifting the cane from the ground when they felt so.

### Data Acquisition and Treatment

The methods below have been used in similar published studies (Sozzi et al., 2012, 2017; Schieppati et al., 2014). Briefly, for each protocol, each subject provided a series of 60 trials per direction of haptic shift (Touch → NoTouch, T-NT; NoTouch → Touch, NT-T). The two protocols (finger–cane) were spaced by a week or more. The order of conditions was randomized across the subjects. The 60 trials were acquired by performing a series of 10 successive acquisition epochs of 240 s each (for both the ‘finger touch’ condition and the ‘cane touch’ condition). Between these acquisition epochs rest periods were allowed, during which subjects were free to sit or move around. Each epoch contained six haptic changes in which the finger touched the pad, or the cane was lowered onto the ground (NT-T), and six changes in which the finger was withdrawn from the pad or the cane was lifted from the ground (T-NT). Each acquisition epoch was divided in periods of 30 s duration centered on the change in haptic condition at *t* = 15 s (**Figures 1C,D**). The exact time instant of the haptic shift was identified on the signal recorded by the strain-gauge of the pad on which the finger was leant or by the force signal recorded by the platform on which the cane was lowered. The abrupt rise in the force signals marked the contact of the finger or of the cane with the stable surface, a brusque return to zero marked the instant of finger or cane detachment. Then, equal-condition trials were aligned at the instant of the haptic shift and averaged. The big trial numbers (*n* = 60 per haptic shift direction: NT-T and T-NT, cane and finger) were necessary in order to get consistent mean values for body oscillation, and to estimate reliably the time following the shift in haptic information, at which body sway modifications occurred. Repetition rate, and rest periods, would not have affected the stabilizing effects of the haptic cue, since stabilization had been observed during both continuous and intermittent light finger touch (Johannsen et al., 2014).

### Levels of Body Oscillation With and Without Haptic Cue

For every trial recorded in each subject, the mean A-P and M-L oscillations of the CoP were computed under all haptic (NT and T, cane and finger) conditions at steady state. These mean oscillation values were calculated on the first and last 10 s periods of each trial period. In this way, the steady state periods did not contain the 5-s intervals just before and just after the sensory shift, and were considered to be stationary and unaffected by the sensory shift (Sozzi et al., 2011, 2012; Honeine et al., 2015).

### Mean Latency of the Changes in Body Sway Oscillations Following the Sensory Shifts

For each subject and condition of haptic shift (addition or withdrawal, both for finger and cane touch), we assessed the latency following the sensory shift at which the CoP oscillation started to diminish, or to increase, depending on the haptic-shift direction (NT-T or T-NT). The latency was estimated on the averaged CoP traces (*n* = 60), centered on the sensory shift. These latencies were assessed only for the frontal plane, because under tandem stance the presence or absence of haptic information produced a much larger gap in the oscillation level in the M-L than in the A-P direction. This allowed a secure application of the statistical procedure used to detect the time at which the oscillations began to change.

In order to estimate the time instant at which the change in haptic condition began to affect the CoP oscillation, each successive value of the averaged trace following the instant of the sensory shift was compared to the mean value of the trace computed during the 15 s before the haptic shift by one-sample Student’s *t*-test with *n* = number of repetitions. The time-interval after the shift, at which the *t*-value of the successive comparisons bypassed the critical value corresponding to a 0.05 probability (one-tailed *t*-test) and remained above that value for at least 100 ms, was taken as the time at which the presence or absence of the haptic information began to affect the postural control mode (Sozzi et al., 2012; Schieppati et al., 2014).

### Time to Reach Steady State Condition

After the initial change in CoP oscillation, the averaged CoP value gradually reached a new steady state pertaining to the new sensory condition. The trace representing the time-course of the CoP oscillation was fitted for each subject and condition with an exponential model (*y* = A + B e^-t/τ^) by the Excel^®^ Solver Utility (Sozzi et al., 2011, 2012; Schieppati et al., 2014). The parameter τ of the exponential model was the time constant of the recovery, A was the value at steady state, and A+B the intercept with the ordinate. A, B, and τ were computed by using the minimum sum squared algorithm by the iterative conjugate gradient method of the Solver utility. The curve of the mean CoP oscillation was fitted from t = latency of changes after the haptic shift until the end of the 30 s time window.

### Statistical Analyses

The mean levels of CoP oscillation calculated at steady state were compared by a 3-way repeated-measure ANOVA with direction of oscillation (M-L and A-P), cane or finger touch condition and the presence or absence of haptic information (NT or T) as independent factors. Two 2-way repeated-measure ANOVAs with cane or finger touch condition and direction of haptic shift (NT-T or T-NT) were used to compare the mean latencies of the change in M-L CoP oscillation level after the sensory shift and to compare the mean time constant (τ) to reach the steady state condition. All *post hoc* comparisons was made with the Tukey’s HSD test. Where the differences were significant, the Cohen’s *d* effect sizes were also reported in order to highlight the strength of the difference (with *d* = 0.2, 0.5, and 0.8 considered as small, medium and large effect size, respectively). The mean forces exerted by the cane or by the finger were compared between ‘Finger touch’ or ‘Cane touch’ condition by the paired Student’s *t*-test, and the Cohen’s *d* effect size was calculated. The software package used was Statistica (StatSoft, United States).

## Results

**FIGURE 2:**
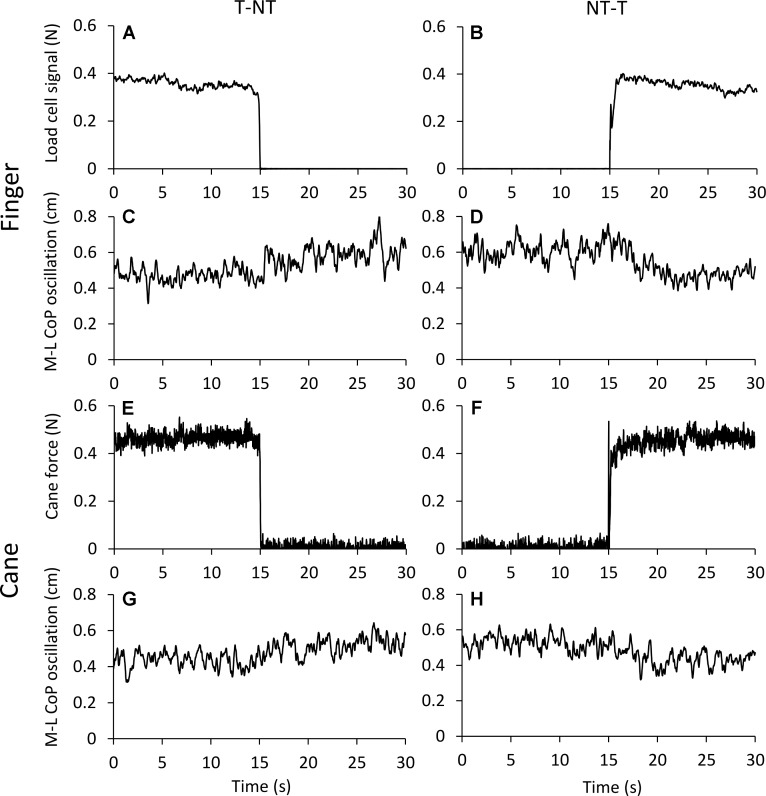
**(A–D)** Average traces (*n* = 60) recorded from one subject under the ‘finger touch’ protocol. **(E–H)** Average traces recorded from the same subject as in **(A–D)**, but under the ‘cane touch’ protocol. The forces exerted with the finger **(A,B)** and the cane **(E,F)** were less than 1N. After withdrawal of the haptic information from finger **(C)** or cane **(G)** the values of M-L CoP oscillation increased at a short delay from the instant (*t* = 15 s) of the shift in haptic condition. When the haptic information entered, both by finger **(D)** and by cane **(H)** contact, the values of M-L CoP oscillation diminished in amplitude.

**Figure 2** shows the averaged traces of the signals recorded in one representative subject standing on the force platform during the T-NT (left panels) and NT-T (right panels) haptic shifts for the finger touch (**Figures 2A–D**) and cane touch (**Figures 2E–H**) protocols. During the touch periods, the force exerted by the subject by means of the finger or by the cane was always less than 1N (**Figures 2A,B,E,F**). When subjects lifted the finger from the touch pad (**Figure 2A**) or lifted the cane from the ground (**Figure 2E**), the values of the oscillations (**Figures 2C,G**, respectively) increased after a short delay from the instant of the sensory shift. Conversely, after the NT-T shift [finger on the touch pad (**Figure 2B**) or cane lowered onto the ground (**Figure 2F**)], the values of the M-L CoP oscillations (**Figures 2D,H**) diminished in amplitude. All subjects referred that when they touched the touch pad or lowered the cane on the ground they felt more stable than in the absence of the haptic reference.

### Body Sway Under Steady State Condition

**FIGURE 3:**
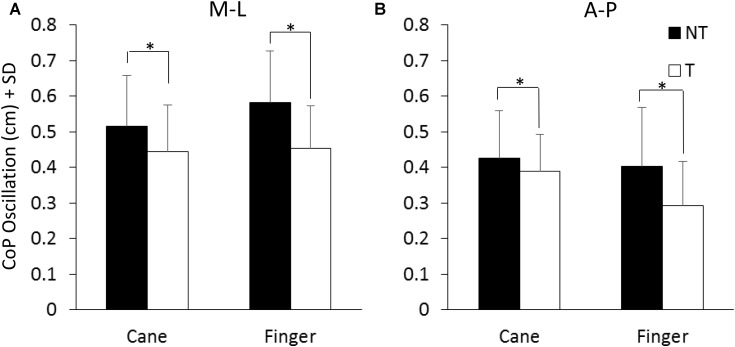
Mean levels of M-L and A-P **(A,B)** CoP oscillation calculated at steady state under NT (black bars) and T (white bars) condition. The oscillation was greater along the frontal (M-L) than the sagittal (A-P) plane and greater under NT than T condition. ^∗^Indicates a significant difference (*p* < 0.05).

**Figure 3** shows the mean values of the CoP M-L and A-P oscillations calculated at steady state, with (T) or without (NT) the haptic information. The oscillations (finger and cane condition collapsed) were larger along the M-L (left panel) than the A-P (right panel) direction [*F*(1,13) = 12.41, *p* < 0.05; Cohen’s *d* = 3.92] during both NT and T conditions. Oscillations were larger in both M-L and A-P directions during the NT (black bars) than during the T period (white bars) [NT vs. T; *F*(1,13) = 27.57, *p* < 0.001; *d* = 5.8]. There was no difference in the CoP oscillations (M-L and A-P collapsed) between cane touch and finger touch conditions [Cane vs. Finger; *F*(1,13) = 0.41, *p* = 0.53]. There were significant interactions between NT or T condition and cane or finger touch condition [*F*(1,13) = 15.41, *p* < 0.05; *d* = 4.33] and between direction of oscillation (M-L or A-P) and cane or finger touch condition [*F*(1,13) = 5.39, *p* < 0.05; *d* = 2.56]. There were no interactions between direction of oscillation and NT or T condition [*F*(1,13) = 1.18, *p* = 0.29] and between direction of oscillation, cane or finger touch condition and NT or T condition [*F*(1,13) = 1.6, *p* = 0.23]. The *post hoc* test showed that the oscillations were always larger under NT than T condition (*p* < 0.001, *d* > 0.6 for all four comparisons), and were slightly smaller in the M-L direction when holding the cane without touching the ground than during the finger no-touch condition (*p* < 0.001, *d* = 1.1).

**FIGURE 4:**
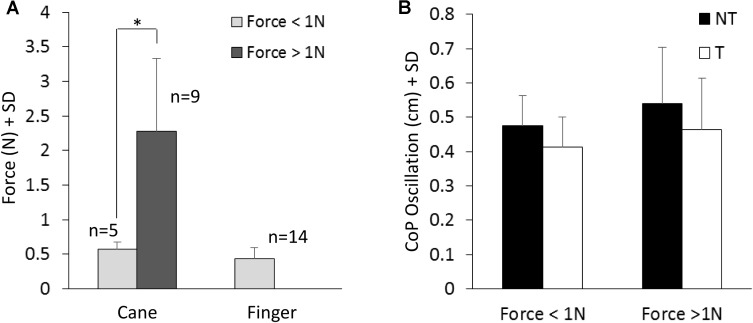
**(A)** Mean value of force exerted by cane or finger during the touch period. The dark column refers to the subjects that exerted more than 1N force. **(B)** Shows the mean M-L CoP oscillations in the two sub-group of subjects **(A)** standing with the cane during NT (black bars) and T (white bars) periods. The oscillations during T (cane) are not related to the level of force exerted onto the ground.

**Figure 4A** shows the mean values of the force exerted by the cane or by the finger during the touch periods and the mean values of M-L oscillations of the CoP (**Figure 4B**) at steady state, with (T) or without (NT) the haptic information. All subjects collapsed, there was a difference between the force exerted by the cane and the finger (paired *t*-test, *p* < 0.05; *d* = 2.13), because nine subjects did not succeed in maintaining a force contact of the cane with the ground below 1N (mean force was 2.3 N ± 1.05, dark gray bar in **Figure 4A**), even if the force exerted by their finger was always smaller than 1N. In the other five subjects (light gray bar), the cane exerted less force, and there was no difference between the force applied by the cane to the ground (0.58 N ± 0.09) and that applied by the finger to the touch-pad (0.45 N ± 0.16) (paired *t*-test, *p* = 0.17). Of note (**Figure 4B**), there was no difference in the mean levels of CoP oscillation while standing with a cane between the two groups of subjects applying a lower or a higher force level (white bars in **Figure 4B**, *t*-test, *p* = 0.51).

### Latencies of Changes in M-L CoP Oscillation Following the Sensory Shift

The latencies of the changes in body sway on touching the ground with the cane or the solid surface with the finger (or vice versa, lifting the cane off ground or removing the finger from the touch-pad) were estimated for each subject on the mean M-L CoP oscillation trace.

**FIGURE 5:**
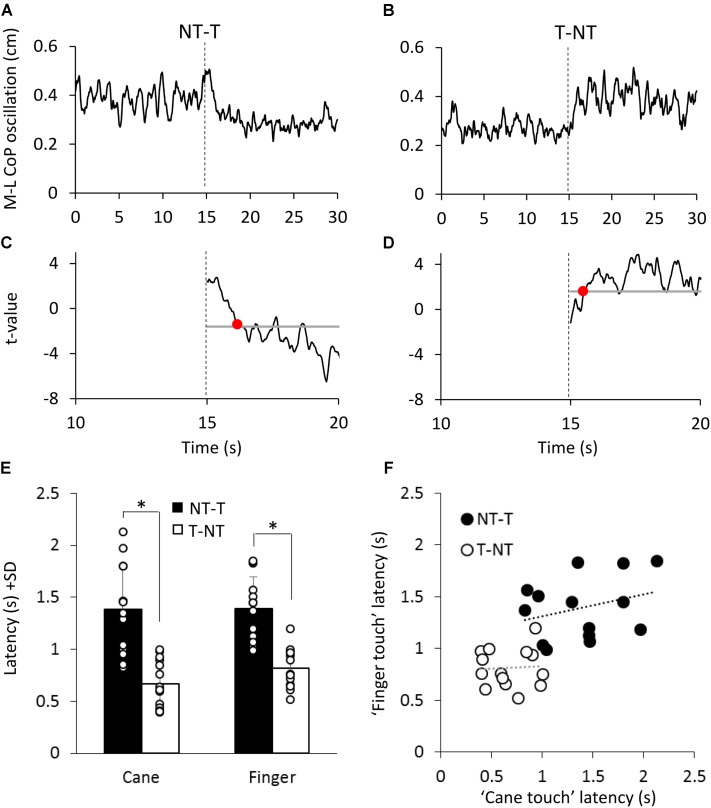
**(A,B)** Mean M-L CoP oscillation of one subject during NT-T and T-NT haptic shift (dotted line at 15 s) under the ‘cane touch’ condition. **(C,D)** The traces show the *t*-values calculated by comparing all the successive mean values of oscillation with the mean value of all pre-shift period. The horizontal line indicates the critical t value corresponding to α = 0.05 probability. The time at which the *t*-value bypassed the critical value and remained above or below it for at least 100 ms (red dot in **C,D**) was set as the latency at which the haptic shift began to significantly affect the M-L CoP oscillation. **(E)** Mean time intervals from the haptic shift to the change in CoP oscillation. These latencies were longer for the addition (NT-T, black bars) than for the withdrawal (T-NT, white bars) of the haptic information. There was no difference in latency between cane touch and finger touch condition. **(F)** For each subject, the latency calculated for the ‘finger touch’ condition is plotted against the latency of the ‘cane touch’ condition both for the NT-T (black circles) and for T-NT shift (open circles). Across subjects, there was no significant relationship between the latencies of cane and touch within condition. ^∗^Indicates a significant difference (*p* < 0.05).

As shown in **Figures 5A,B**, at a short delay following the shift in haptic condition (*t* = 15 s), the oscillations decreased when the haptic information from cane or finger was available (NT-T condition) or increased when the haptic information was removed (T-NT condition). Latencies of these changes were estimated by comparing each mean oscillation value after the haptic shift with the mean oscillation value calculated for the 15-s period before the shift (**Figures 5C,D**).

**Figure 5E** shows that, across subjects, for the NT-T haptic shift, mean latencies ranged from 0.96 s to 2.1 s for the cane-touch condition and from 0.99 s to 1.8 s for the finger-touch condition. For the T-NT shift, latencies ranged from 0.4 s to 1 s for the cane and from 0.5 s to 1.2 s for the finger condition. In the **Figure 5**, the mean latencies across subjects for the two sensory shifts (NT-T and T-NT) and for the two touch conditions are also reported. There was no difference in the mean latencies between the cane-touch and the finger-touch condition [*F*(1,13) = 1.59, *p* = 0.23]. There was a difference between NT-T and T-NT condition, since latencies were longer for addition than withdrawal of haptic information [*F*(1,13) = 61.29, *p* < 0.001; *d* = 6.25] and there was no interaction between cane or finger touch condition and haptic shift direction [*F*(1,13) = 0.79, *p* = 0.39].

Some subjects were equally slow in responding to both cane and finger input, and some fast on both cane and finger input. On the other hand, all subjects were equally fast on withdrawing the haptic input, from either source. However, there was a large variance in the latencies. Within each haptic shift (NT-T or T-NT), the relationships between the cane and finger latencies were not significant (linear regression for T-NT: *y* = 0.04*x* + 0.78, *R*^2^ = 0.003, *p* = 0.85; for NT-T: *y* = 0.21*x* + 1.09, *R*^2^ = 0.08, *p* = 0.3). The difference between the values of the intercepts with the ordinate of the two linear regressions was similar to the difference between the mean latencies of the NT-T and T-NT conditions. All data points collapsed, there was a good relationship between finger and cane data (*y* = 0.52*x* + 0.57, *R*^2^ = 0.44, *p* < 0.001, regression line not drawn in the **Figure 5**).

Across all subjects, we found no significant relationship between latencies (in any condition) and age at first diagnosis of visual impairment (*p* > 0.16 for the slope of the regression lines drawn for all four conditions). Neither was there any significant regression between latencies and the number of years that subjects had been using the cane at the time of this study (*p* > 0.45 for the slope of the regression lines drawn for all four conditions), in spite of the ample year range (9.9 years ± 9.7).

### Time Course of the Changes in M-L CoP Oscillation

**FIGURE 6:**
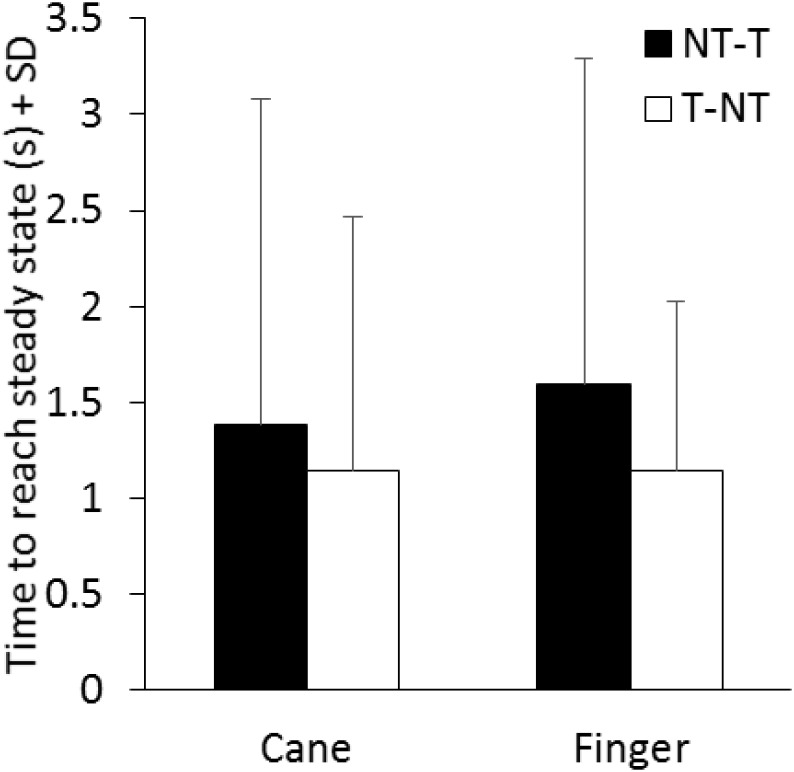
Mean time constants of the recovery of the oscillations to a new steady state pertaining to the new sensory condition. There is no significant difference in the time constant between conditions (cane or finger touch) and between directions of haptic shift (NT-T or T-NT).

The time necessary to gradually reach a new steady state (time constant, τ) after the earliest detectable changes in body sway connected with the sensory shift was also estimated for each subject, based on the time course of the mean M-L CoP oscillation trace. In the case of the NT-T, after the latency period from the instant of the shift, the M-L CoP oscillation decreased until it reached a new steady state (for both cane or finger touch), vice versa after the T-NT shift. The mean time constant calculated for the two haptic shifts under both cane and finger touch condition are reported in **Figure 6**. There was a large τ variability across subjects under all conditions. There was no difference between the time constants of cane and finger touch condition [*F*(1,13) = 0.08, *p* = 0.78], no difference between directions of haptic shift [NT-T vs. T-NT: *F*(1,13) = 1.84, *p* = 0.19] and no interaction between cane or finger touch condition and haptic shift direction [*F*(1,13) = 0.12, *p* = 0.74].

## Discussion

In standing blind subjects, we investigated the stabilizing effect produced by the contact of the cane with the ground and those produced by a fingertip touch of a firm surface. We also estimated the promptness of balance stabilization (or destabilization) on adding (or withdrawing) the haptic inputs. We found that both the contact of the finger with the cue surface and the contact of the cane with the ground reduced the amplitude of oscillation of the CoP to a similar extent. The delay to the change in body oscillation on reaching the haptic surface was significantly longer for addition than withdrawal of the haptic information, but was not different between the two haptic conditions. The findings show that using a cane provides an aid to enhance body steadiness, as effective as a finger touch, and that both haptic cues are processed by the nervous system within an equal time interval.

Haptic information derived from fingertip or cane contact with a surface, even with a level of contact force not granting mechanical stabilization (Jeka et al., 1996; Lackner et al., 2001; Kouzaki and Masani, 2008; Sozzi et al., 2012, 2017), is sufficient for consistently reducing body sway. The haptic cues arising from cutaneous, kinesthetic, and proprioceptive sense (Lederman and Klatzky, 2009) provide useful information in terms of specifying body position in space much as vision (Peterka, 2002; Honeine et al., 2015). Haptic information can substitute for the absence of vestibular (Bernard-Demanze et al., 2015), visual (Jeka et al., 1996; Sozzi et al., 2012; Honeine et al., 2015) or somatosensory information (Kotecha et al., 2012) in the control of balance and gait. Haptic cues improves postural control in the elderly (Baccini et al., 2007; Albertsen et al., 2012), and can even help Parkinsonian patients during stance and walking (Rabin et al., 2013, 2015). Bolton et al. (2011) revealed a facilitation of the late cortical response to electrical stimulation of a nerve from the hand during haptic balance tasks. They suggested that task-specific regulation of the cortical representation of fingertip afferent input occurs when it is relevant to providing stable cues for balance control. These findings imply the relevance of the haptic input and the complexity of the changes in the cortical excitability induced by the haptic stabilization during stance. The latency from the haptic cue to the onset of balance stabilization more than accounts for the delays connected with supraspinal, possibly cortical processing (Tokuno et al., 2009; Onishi et al., 2010; see for a discussion, Sozzi et al., 2017).

In blind subjects, haptic information normally originates from touching a solid surface with the hand or fingers, or from using a tool, normally a cane. This can serve for mechanical support of the body (Maeda et al., 1998; Bateni and Maki, 2005; Virgili and Rubin, 2010), for exploration of the shape of the walking path or ground texture (Nunokawa and Ino, 2010; Ranavolo et al., 2011), or for referencing the body to the ground and to the vertical in the absence of vision (Jeka et al., 1996; Albertsen et al., 2012; Lacquaniti et al., 2015). Here, we were interested in comparing the extent and the promptness of the balance stabilization by finger touch to a cue-surface close to the body and by cane contact to the ground in visually impaired subjects, in search of a difference connected to the different features of the haptic tasks.

### Cane and Finger Touch Stabilization

In this population of adult blind subjects, standing in tandem Romberg position eyes closed, we found that the effects on body sway exerted by the haptic information either from the cane or from the fingertip was undistinguishable. This were true also for the two subjects with visual acuity < 2/10. In passing, all these subjects used their preferred hand for reaching with the cane or finger much as sighted subjects do (Stone and Gonzalez, 2014), indicating that blindness did not affect the way deliberate movements in search of stabilizing sensory cues are controlled.

Not only the extent of stabilization under steady-state sensory condition was similar, but the latency of the changes in body sway on both adding or withdrawing the haptic information from either source (cane, finger) was similar as well. Further, the time course for the body sway to reach the level corresponding to the given postural condition (with or without the haptic input) was also similar, suggesting no changes in the integration and re-weighting processes of haptic cues from cane and finger.

What matters in the postural stabilization seems to be the presence (or absence) of the haptic information itself rather than the source of the haptic input or the way it is achieved. A contact far from the body, like that from the cane tip, with the arm somewhat flexed and abducted, assists balance as much as the touch with the tip of the index finger, with the hand placed very close to the body. Further, the possible prevalence of the coarser proprioceptive input in the case of the cane than the finger (Buchthal, 1982; Schieppati and Ducati, 1984; Phillips and Johnson, 1985) did not seem to count. Broadly similar stabilizing effects between different passive tactile cues had been described by Rogers et al. (2001).

We also noted that some subjects exerted more than 1 N force with the cane on the force platform, but that the level of their stabilized sway was indistinguishable from that observed when the same subjects exerted less than 1 N with the finger or from that of the subjects that exerted less than 1 N with the cane. Different forces would produce a different afferent input, but either the differences are negligible, or it is the mere haptic information from the environment that matters. As to the level of force exerted, others have shown in sighted subjects that standing while lightly or forcefully touching a wall does not produce significant difference in body sway between the two touching conditions (Watanabe et al., 2010; Ustinova and Langenderfer, 2013; Baldan et al., 2014). Similar results were obtained by Jeka et al. (1996): they asked the subjects (sighted and blind subjects) to maintain the tandem Romberg stance without the cane or while holding the cane in a perpendicular or slanted orientation and to apply two different level of force (<2 N or as much force as they desired). They found that the postural sway attenuation was greatest with a slanted cane, irrespective of the level of force applied.

### Time Course of Haptic Stabilization

The latency and time-course to stabilization (or destabilization on withdrawing the haptic input) were also similar between cane and finger touch. This would imply that lowering (or raising) of the cane by a complex coordinate movement entailing a postural adjustment (although minor, given the light weight of the cane) does not produce a longer latency than the simple lowering (or raising) of the index finger. Since the instruction was to exert a very low force level during the task, we were expecting a stronger control during cane than finger lowering to the haptic surface, possibly accompanied a greater cognitive effort that would have delayed the integration process (Honeine et al., 2017). Again, what matters seems to be the contact itself, producing the sensory haptic volley.

Of course, a sizeable difference in latency was observed, as expected, between addition and subtraction of the sensory input. The origin of this difference has been discussed at length in previous papers (Sozzi et al., 2011, 2012; Schieppati et al., 2014; Honeine et al., 2017). We found here that there was no significant interaction between cane and finger or addition and withdrawal of the haptic stimulus. The control of the tool and the calibration of its contact force, which would have been expected to require a greater attention cost than the finger’s (Williams et al., 1998; Saradjian et al., 2013; Johannsen et al., 2014), was apparently not critical to the processing of the haptic information. Since attention can effectively modulate tactile change detection (Spence and Gallace, 2007; Van Hulle et al., 2013), we would also argue that under this challenging stance condition the same level of attention was likely devoted to both finger touch and cane haptic input. The brain is obviously capable of detecting meaningful haptic transients, regardless of the sensory channel through which these are conveyed (proprioceptive, tactile) and of the concurrent motor action. The peripheral receptors are designed for this. And the brain anticipates the transition (Chapman, 1994), contributing to enhanced performance during both cane and finger touch, by preparing the postural centers to assign the appropriate functional value to the relevant haptic input (Schieppati and Nardone, 1995). This process may be the same as that observed in the visual stabilization of posture in sighted subjects (Sozzi et al., 2011, 2012) pointing again to the similar processing of stabilizing cues from different modalities.

### Comparison to Sighted Subjects’ Haptic Stabilization

In this population of blind subjects, the extent of the stabilization in response to cane or finger touch was similar to that of a population of sighted subjects studied previously (Sozzi et al., 2012, 2017). The latency, at which the body sway began to diminish in response to the haptic input (or to increase after its withdrawal) and the time-course for the body sway to reach the steady-state level (with or without the haptic input) were also similar to that described in sighted subjects.

It appears that blindness did not confer any advantage to the processing of the haptic input, from either cane or finger, toward a better or more prompt stabilization of balance. These considerations do not necessarily run counter to the hypothesis that blind subjects recruit visual-related cortices to process information from other perceptual sensory modalities through cross-modal plasticity (Lewis et al., 2010; Voss et al., 2016), or that adapted sensory-motor functions take place in multimodal integration regions (Easton et al., 1998; Ortiz-Terán et al., 2016). It is not clear whether new circuits are being recruited more promptly during the haptic-induced postural stabilization in low-vision subjects. What is known already is that body sway in blind and low-vision subjects is not lesser than in sighted subjects eyes closed (Schmid et al., 2007; Russo et al., 2017), indicating no superior capacity of exploiting the cutaneous input from the foot sole (Roll et al., 2002) or finger (Bolton et al., 2011) or the proprioceptive inflow from the postural muscles (Schieppati and Nardone, 1999; Marchand-Pauvert et al., 2005). The level of sway reached on obtaining the haptic cues (from either cane or finger touch) is also similar to that of sighted subjects. Under different experimental conditions, testing whether visual and haptic map learning yield functionally equivalent spatial images in working memory, Giudice et al. (2011) have shown no reliable differences between sighted and blind subjects for orientation and turning time measures, and suggested that the equivalent behavior was mediated by an amodal spatial image. Kim et al. (2014) found equivalence of sighted and low-vision subjects in the perception of tactile stimuli in terms of haptic perception and user interface needs, and suggested that the everyday use of residual visual capacities was less likely to have enhanced their haptic capabilities via brain plasticity. One of the reasons for not finding the expected edge in low-vision subjects might be connected to their inability to exploit the enhanced tactile acuity, which is normally enabled by vision in sighted subjects (Kennett et al., 2001; Serino and Haggard, 2010; Konen and Haggard, 2014; Colino et al., 2017). Even in sighted subjects, when vision is not available, proprioceptive information from the support can substitute for vision (Krishnan and Aruin, 2011). All in all, it seems that the basic mechanisms whereby stance support modulates the ‘postural set’ (Ivanenko and Gurfinkel, 2018) and reduces body postural oscillations are common to both blind and sighted subjects.

### Limitations in the Assessment of the Latency to Sway Changes

As far as the rapidity with which the haptic input is integrated in the control of standing balance, the picture is not unequivocal. In a previous communication, we found that the latency to body sway change on touching a firm surface with the finger was somewhat shorter in a very small cohort of early blind subjects than in late blinds (Schieppati et al., 2014). That finding was suggested to be a sign of learning to re-weight the haptic cues in order to obtain a rapid integration of the stabilizing input, possibly connected with the brain plasticity in the early years of these subjects (Dormal et al., 2012). Those short latencies did not recur in the presently studied population. The latencies of the subjects whose vision became severely impaired early in life (3/14) is within the range of the entire population of low-vision subjects. Therefore, we would be reluctant in shedding any firm divide between congenital or early blind subjects and late blind subjects or subject with severely impaired vision, based on the extent of the stabilization (see also Soares et al., 2011) or on the latency of their postural adaptation to haptic input.

We would point out again (Schieppati et al., 2014) that our method of estimating latencies is based on statistical assumptions and is affected by the number of trials averaged, the individual mean level and variability of the center of pressure oscillation during the trials and the criteria set for the assessment. There must have been a change at the CNS level prior to the value determined by a significant *t*-test, but this statistical procedure cannot detect the ‘true’ time at which a change in the balancing pattern occurs. These uncertainties, though, would have affected to the same extent the measures taken in both the cane and finger condition or, for that matter, in both blind and sighted subjects. Moreover, under conditions where repeated trials are being performed, the learning ratio of different subjects of different cohorts might affect the outcome (Postma et al., 2007). Fortin et al. (2008), studying navigational proficiency in blind persons, found no differences in their superior skills and hippocampal volume, regardless of their blindness being congenital or acquired. Thus, whether or not brain plasticity in the early blindness confers an advantage in the capacity to use egocentric haptic information (Postma et al., 2007; Ruggiero et al., 2012) and to exploit it for balance stabilization would require further investigations in larger cohorts of subjects. In this context, we note that further understanding of neuroplasticity would be welcome because of its casual role in the embodiment of neural prostheses (Lebedev and Nicolelis, 2006).

### Perspectives

Recent studies demonstrated that a vibratory noise applied to the fingertip while standing with eyes closed and touching a solid surface improved postural stability more than merely touching the surface (Magalhães and Kohn, 2011a,b). Whether adding a concurrent sensory stimulation through a different modality channel can also diminish the latency to integrate the haptic input into the balance control process in low-vision subjects is an open question worth investigating (see Lebedev and Ossadtchi, 2018, for a general discussion about instructing the brain to improve learning of haptic feedback).

Various devices have been developed to aid blind or visually impaired subjects in avoiding obstacles during walking (Tzovaras et al., 2004; Penrod et al., 2005; Nunokawa et al., 2014; Buchs et al., 2017; see for a review Pawluk et al., 2015). For instance, ultrasonic or infrared sensors were mounted on the cane in order to estimate the distance between the user and an obstacle, or to judge hardness of an object. Instrumentation of the cane, taking into account the time necessary to integrate haptic information, could help blind subjects to feel more stable and more confident during the activities of everyday life including gait, since dynamic stability is reduced in low-vision subjects (Hallemans et al., 2010). The latency to stabilization in response to haptic input should also be considered when designing devices to help visually impaired subjects orienting themselves while stepping along a path unbeknownst to them but with guide lines detectable by an instrumented cane (as for instance in Hirahara et al., 2006).

## Conclusion

The haptic input can and does stabilize balance under challenging conditions, such as the tandem Romberg posture. The haptic effect is broadly similar regardless of the source of the information, finger or cane. The haptic input is given priority by the brain, independently of task differences while aiming at the haptic target. Drawing from findings by Rabin and Gordon (2004, 2006) obtained in a different perspective, one would argue that the spatially meaningful tactile cues and the proprioceptive feedback from the entire upper limb are integrated based on the specific task priority of the current task.

There does not appear to be differences between these blind subjects and the sighted subjects studied in a previous recent investigation (Sozzi et al., 2017). The similarities between cane and finger effects on balance stabilization and the similar behavior of low-vision and sighted subjects are expression of a normal processing of haptic input in low vision, and constitute a rationale for inclusion in their rehabilitation (Meyniel et al., 2017) of orienteering and training course with emphasis on the use of the cane (Kimura et al., 2012). The similarities of cane and finger input and the effectiveness of cane contact would also warrant use of the cane and instrumentation of it in order to enhance the sensory feedback when necessary, as when postural orientation is modified (Bisdorff et al., 1996) or when aging degrades haptic sense (Kalisch et al., 2008; Giudice et al., 2017).

## Author Contributions

SS contributed with data collection, data analysis and drafted parts of the manuscript. FD and MoS recruited and diagnosed the patients. OC contributed with data collection and data analysis. MaS contributed with project creation, data analysis, and wrote and edited the manuscript.

## Conflict of Interest Statement

The authors declare that the research was conducted in the absence of any commercial or financial relationships that could be construed as a potential conflict of interest.
